# Exome sequencing contributes to identify comorbidities in a rare case of infant ARDS induced by the *CD40LG* mutation

**DOI:** 10.1186/s12920-022-01303-y

**Published:** 2022-07-08

**Authors:** Xue Gong, Yunru He, Guoyan Lu, Yulin Zhang, Yu Qiu, Lina Qiao, Yifei Li

**Affiliations:** Key Laboratory of Birth Defects and Related Diseases of Women and Children of MOE, Department of Pediatrics, West China Second University Hospital, Sichuan University, No. 20, 3rd Section, South Renmin Road, Chengdu, 610041 Sichuan China

**Keywords:** ARDS, Genomic sequence, X-linked hyper IgM syndromes, Immune deficiency

## Abstract

**Background:**

Acute respiratory distress syndrome (ARDS) causes significant mortality in young children with certain diseases. Early diagnosis and treatment can reduce infant mortality. Here, we report a rare case of exome sequencing in the early diagnosis of immunodeficiency in an infant.

**Case presentation:**

A four-month-old full-term male infant presented with severe shortness of breath, hypoxemia, and unexplained parenchymal lung lesions. A series of examinations were performed to search for potential culprit viruses but negative results were obtained with the only exception being the rhinovirus that tested positive. The child’s family history revealed he had a brother who died of severe infection at the age of two years. We performed an exome sequencing analysis and a mutation analysis of *CD40LG* to obtain genetic data on the patient. Besides, we used flow cytometry to measure the *CD40LG* expression levels of activated T cells. A retrospective review of all the *CD40LG* mutant-induced X-linked hyper IgM syndromes (XHIGM) had been conducted to assess the differences between clinical and genetic molecular features. Finally, a regular intravenous immunoglobulin (IVIG) regimen led to steady breathing, the correction of hypoxemia, and a progressive improvement of lung CT scans. During follow-up, the patient received an IVIG regimen and his CT images improved. Moreover, his parents took advantage of pre-implantation genetic testing with in vitro fertilization to have a healthy twin offspring who did not carry such a mutation according to the early exome sequencing for the proband. Compared with other *CD40LG* mutant cases in our center, this proband displayed a normal plasma immunoglobulin level and he should be the youngest infant to have a molecular diagnosis of XHIGM.

**Conclusion:**

Usually, XHIGM would not be suspected with a normal plasma immunoglobulin concentration. However, as we could not identify a potential comorbidity or risk factor, exome sequencing helps target this patient's real facts. Thus, this case report calls for exome sequencing to be performed in the case of unexplained infections when immunodeficiency is suspected after general immunological tests, especially for cases with a contributive family history among infants as the maternal transfused immunoglobulin might mask immune deficiency.

**Supplementary Information:**

The online version contains supplementary material available at 10.1186/s12920-022-01303-y.

## Background

Acute respiratory distress syndrome (ARDS) is a clinical syndrome characterized by a distortion of the alveolar epithelial-endothelial barrier’s permeability, which is not related to cardiogenic pulmonary edema [1]. Injuries usually occur within the pulmonary capillary endothelium either as a result of localized lung infections or as secondary effects of systemic inflammation associated with, for example, sepsis or pancreatitis [2–4]. In 2015, a specific definition for pediatric ARDS was coined at the Pediatric Acute Lung Injury Consensus Conference [5]. The differences between adult and pediatric ARDS lie in its causes and in clinical practice, with the morbidity and mortality rates among young children being higher. Pediatric ARDS is a severe condition and one of the leading causes of ICU deaths, warranting further research to explore the associated risk factors, comorbidities, precise treatment approaches, and outcome predictors [6, 7].

A previous review revealed that there has been considerable focus on understanding the conditions that predispose patients to this disease [8, 9]. Furthermore, physicians seek to delineate the relationship between ARDS risk factors and its clinical outcomes. Apart from the risk factors for ARDS, comorbidities also strongly contribute to the development and severity of ARDS [6, 7, 10]. Among these comorbidities, congenital and acquired cardiac lesions are the most widely recognized factors. Immune suppression or immune deficiency has been identified in less than 10% of young patients with ARDS [11]. However, the associated mortality rate secondary to immune dysfunction is 46%, which is considerably higher than that associated with prematurity (19%) and congenital heart diseases (11%) when a timely diagnosis, which contributes to precise management, is not forthcoming [6, 7].

Children with immune deficiencies are always susceptible to opportunistic infections, neutropenia, autoimmune diseases, and malignancies [11]. Studies have found that more than 50% of young male patients develop symptoms at the age of one year and more than 90% do so at the age of four years [12, 13]. The fact that ARDS is not the key symptom of immune deficiency makes the diagnosis of immune deficiency at the onset of ARDS in children challenging in the absence of any other symptoms. Such patients are thereby placed in the high-risk category for recurrent ARDS, which is associated with a significant mortality rate [14]. Consequently, it is essential to consider immune deficiency in cases of unexplained ARDS among young children.

Herein, we report the case of a four-month-old infant with ARDS in whom all potential risk factors and comorbidities had been ruled out. At the same time, we failed to determine any disorder of immune function via basic laboratory tests. Nevertheless, as immune deficiency was suspected, exome sequencing was performed to identify any potential genetic variants. Ultimately, a hemizygous mutation of *CD40LG* was discovered and IVIG administration was scheduled, which led to a satisfactory prognosis for this infant.

## Case presentation

### Cases involved and ethical compliance

A retrospective review of all the *CD40LG* mutant-induced X-linked hyper IgM syndromes (XHIGM, OMIM #308230) had been conducted according to their medical archives. All the patients included should have been diagnosed with a severe infection or immune system disorder at least once. The exome sequencing results and clinical data had been accessed totally. Patients were excluded if the *CD40LG* mutation was predicted as a polymorphism. This study was approved by the ethics committee of the West China Second Hospital of Sichuan University (approval no. 2014-034). For all the participants who were under age of 16, we obtained the written informed consent to participate in this research from all the reported seven patient’s parents before performing exome sequencing and for the inclusion of the patient’s clinical and imaging details in subsequent publications. Besides, written informed consents were obtained from all the involved adults in this study.

### History of illness

**Fig. 1:**
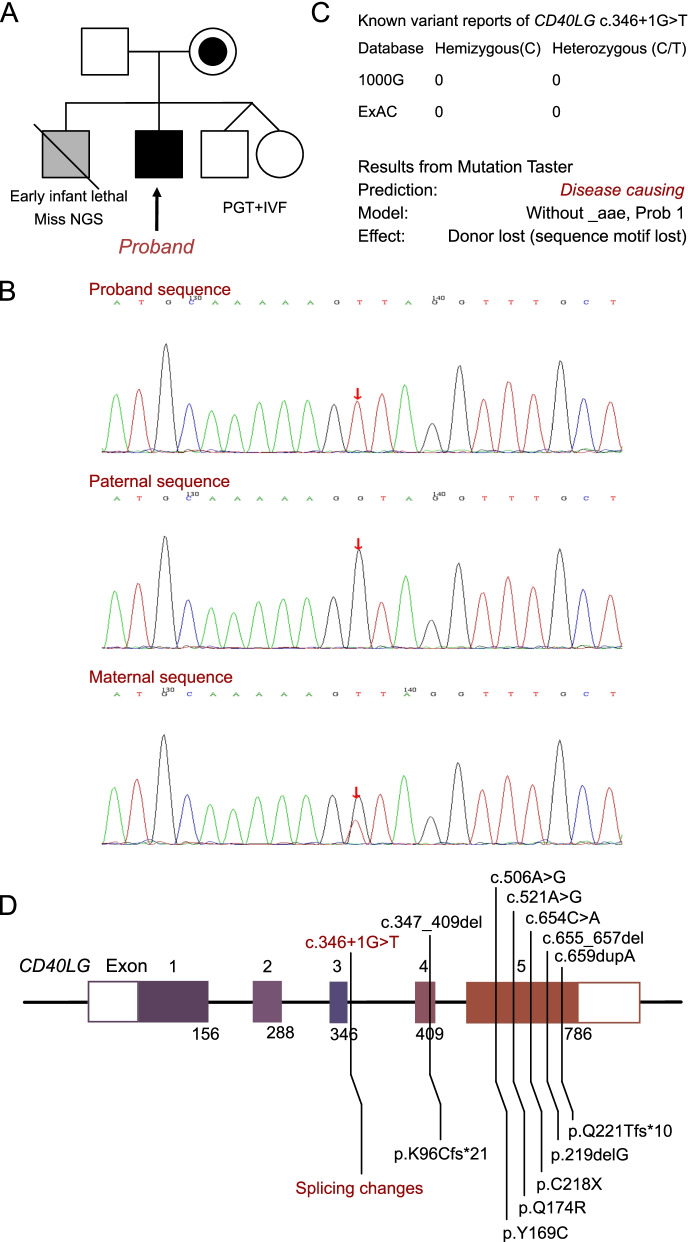
Information on *CD40LG* mutations in this family. **A** The family pedigree reveals that the maternal carrier of *CD40LG* 346+ 1 G > T, and his elder brother died early without exome sequencing. This proband presented infant ARDS with a hemizygous *CD40LG* 346+ 1 G > T mutation. Besides, the next pregnancy of this couple led to the birth of a healthy twin who did not carry the *CD40LG* 346+ 1 G > T mutation. **B** Sanger sequencing validation of this patient and his parents. **C** The reported cases of *CD40LG* 346+ 1 G > T mutation in database. And Mutation Taster predictive result for this mutation. **D** The genetic and protein sequencing variant results of all the seven patients who had a diagnosis of XHIGM with *CD40LG* mutations

This proband is a four-month-old full-term male infant suffering from shortness of breath who was brought to our pediatric intensive care unit. He demonstrated normal growth and development prior to the onset of the disease. The infant developed a violent cough that continued for five days and was diagnosed with bronchitis. The cough was treated with a three-day course of antibiotics despite which the infant presented with severe shortness of breath and hypoxemia that required noninvasive positive pressure ventilation. The parents stated that the infant had no relevant disease history and declined any history of perinatal resuscitation and neonatal ICU hospitalization. Moreover, they reported the death of the infant’s elder brother at the age of two years due to severe pulmonary infection and septic shock without any risk factor (Fig. 1A).

### Laboratory results

The infant’s respiratory rate rose to 70 breaths/min and his arterial oxygen saturation dropped to 83% when nasal oxygen was administered. He responded poorly to external stimuli and was irritable. Scattered bilateral wet rales were perceived in his lungs, and an analysis of his arterial blood gases revealed the following results: pH: 7.312, pO2: 6.07, kPa: pCO2: 4.82 kPa, and BE: − 7.6 mmol/L. The procalcitonin level increased slightly to 0.075 ng/mL. Bacterial cultures performed using his blood and bronchial secretions were also negative. A series of tests were performed to identify potential viral infections, of which only the rhinovirus test result was positive. SARS-CoV-2, influenza A/B, adenoviruses, respiratory syncytial virus, and other viruses associated with respiratory infections were also ruled out, as were mycoplasma and chlamydia. The results of CT scans revealed reduced light transmittance of both lung fields and extensive bilateral areas with diffuse infiltrates, indicating the presence of a parenchymal lung lesion. Ultrasonography revealed pulmonary edema.

**Fig. 2:**
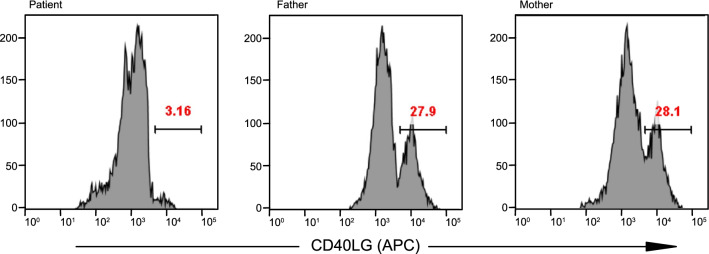
Flow cytometry demonstrated the decreased level (3.16%) of CD40LG+ activated T cells (CD3+ and CD8−) that were expressed (left panel). Also, his father and mother demonstrated similar CD40LG+ levels of 27.9% and 28.1% (middle and right panels), respectively

This case had been considered a case of ARDS without any particular risk factor or comorbidities at infancy. Therefore, immune deficiency was suspected, and general immunological tests revealed that the proportion of subgroups of immune cells was abnormal (the ratio of CD3+ T cells was 41.0% [n.v. 48–75%] and that of T helper cells (CD3+ and CD4+) was 25.5% (n.v. 33–58%), while there was a 50.2% elevation of CD19+ B lymphocytes (n.v. 14–39%) and a 1% reduction of CD27+ lymphocytes (n.v. 4–10%) had been observed. Although the IgA concentration dropped to 0.05 g/L (n.v. 0.1–1.31 g/L), normal volumes of IgG, IgM, IgE, C3, and C4 were recorded (the levels of types of immunoglobulins are listed in Table 1 with the corresponding reference ranges). However, FACS demonstrated the decreased level (3.16%) of CD40LG+ activated T cells (CD3+ and CD8−) that was expressed (Fig. 2, left panel) while his father and mother demonstrated percentages of 27.9% and 28.1% (Fig. 2, middle and right panels), respectively. The detailed protocol for FACS was summarized in Additional file 1.

**Table 1:** The summary of major clinical manifestation with CD40LG mutant X-linked hyper-IgM syndrome cases series in one center

Case No	Mutation site	Model probability value	Variant carrier	Onset age	Diagnosis	Pathogens	Clinical outcome	Family history	Plasma level of IgM	Plasma level of IgA	Plasma level of IgG	Plasma level of IgE
1	c.346+ 1 G > T	1.000	Maternal	2 m	ARDS	Rhinovirus	Alive	An elder brother died due to severe infection	0.83	0.05↓	15.10	13.60
2	c.521 A > G; p. Q174R	0.997	Maternal	15y1m	Sepsis	Pseudomonas aeruginosa	Dead	Negative	4.36↑	0.09↓	0.42↓	14.86
3	c.654 C > A; p. C218X	0.999	Maternal	2y6m	Encephalitis	Toxoplasma gondii	Alive	Negative	3.84↑	0.36	< 0.33↓	< 5.00
4	c.506 A > G; p. Y169C	0.998	Maternal	7y6m	Leukoencephalopathy	Jamestown Canyon virus	Alve	Negative	2.79↑	0.05↓	1.31↓	21.27↑
5	c.347_409del p. K96Cfs*21	0.999	De novo	7y	Recurrent pulmonary infections	–	Alive	Negative	2.59↑	0.08↓	0.46↓	11.12
6	c.655_657delGGG p. 219delG	0.999	Maternal	1y2m	Sepsis	Cytomegalovirus	Alve	An elder brother died due to severe infection	2.12↑	0.07↓	0.37↓	17.20↑
7	c.659 dupA p. Q221Tfs*10	0.999	De novo	1y10m	Recurrent pulmonary infections	Candida	Alive	Negative	1.96↑	0.14↓	0.82↓	< 5.00

^#^Negative values for IgM, 0.21–1.92 g/L; IgA, 0.19–1.31 g/L; IgG, 2.86–16.80 g/L; IgE, < 15 IU/mL

↑ indicated elevated plasma level; ↓ indicated reduced plasma level

### Molecular results

As previously noted, the infant’s family history revealed the death of a two-year-old male sibling from an unknown cause. Therefore, genetic mutations of immune deficiency were strongly suspected, and exome sequencing was performed for this patient. A hemizygous mutation of *CD40LG* at c.346+ 1G > T (splicing-inducing) had been identified. The detailed information on the exome sequencing analysis is found in the Additional file 1: Section. Furthermore, the patient’s mother was a carrier of the variant. The Sanger confirmation is presented in Fig. 1B. According to researchers at the American College of Medical Genetics, this variant is an uncertain one, the same as PVS1 + PS4 + PM2. Further, it has not been reported in any population, and this is the first reported case of this site mutation. An analysis performed using MutationTaster revealed that it was disease-causing (probability: 1, sequence motif lost, Fig. 1C). SPIDEX also revealed a significant impact on RNA splicing (|Z| > 2). The final diagnosis for this patient was pediatric ARDS induced by XHIGM in response to a rhinovirus infection during infancy.

### Treatment and clinical outcome

**Fig. 3:**
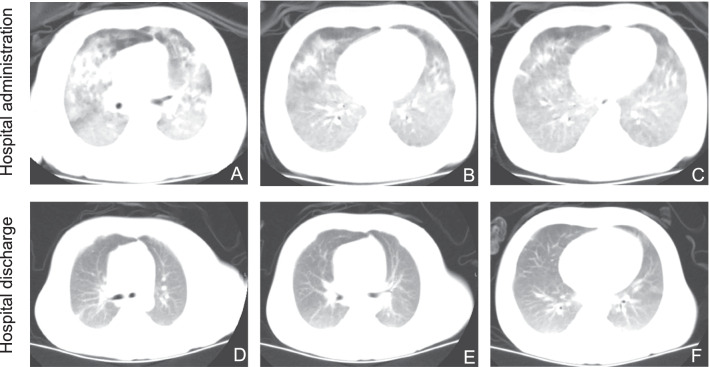
CT scanning images for this patient before and after treatment. **A**–**C** The section images presented diffused bilateral infiltrates and parenchymal lung lesions at the time of PICU administration on the transverse view from the apex level to diaphragm level. **D**–**F** The images present the CT scanning results after treatment before discharge on similar transverse views as the previous one

Immediately after the patient’s admission, a CPAP/BiPAP nasal mask was placed with a FiO2 of 55% to main the SpO2 at approximately 95%. Vancomycin and meropenem were administered. Methylprednisolone was also added to the treatment regimen to reduce systemic inflammation. Intravenous immunoglobulin (IVIG) was administered at a daily dose of 500 mg/kg) for four days during the acute phase of ARDS. After ten days of antibiotic treatment and supportive care, the patient recovered and the non-invasive ventilator was removed. The results of a CT scan and an X-ray revealed a reduction in the bilateral parenchymal diffuse infiltrates (Fig. 3). After that, IVIG administration (1000 mg/kg/month) was scheduled to prevent recurrent respiratory tract infections and facilitate normal development during follow-up.

### Next pregnancy using pre-implantation genetic testing (PGT) + in vitro fertilization (IVF)

Moreover, exome sequencing demonstrated a newly reported variant of *CD40LG* and provided a possible explanation for the ARDS of the proband and the reason for the early death of the first baby. So, according to the genetic background screening, the parents underwent PGT and IVF to have a healthy twin offspring who did not carry such a mutation.

## Discussion and conclusions

It has long been established that preexisting immune deficiency in both adult and pediatric patients increases the risk of ARDS development and is associated with worse post-ARDS outcomes. A previous study reported that 20.8% of adult patients with ARDS were immunocompromised in some way [11]. While human immunodeficiency virus (HIV) infection is the main cause of immunodeficiency among adult patients, genetic mutations are the dominant cause of pediatric immune deficiency. Exome sequencing is currently the gold standard for diagnosing genetically-related immune deficiency and is widely applied given its affordability. Moreover, familial next-generation exome sequencing can be completed within two weeks. Therefore, for a particular condition, exome sequencing is of considerable value for determining whether suspected lesions have a genetic cause, thus facilitating diagnosis and improving long-term outcomes as well as enabling the interpretation of a definitive prognosis.

XHIGM, which accounts for 65–70% of all hyper IgM (HIGM) cases, is the oldest and most common HIGM class, which is caused by mutations in the *CD40LG* gene [15]. Over 75% of patients with XHIGM develop symptoms of immunodeficiency in their first year of life. This gene is located in the long arm of the X-chromosome (26.3–27.1) and contains five exons that are responsible for coding amino acids. CD40L, which belongs to the tumor necrosis factor superfamily, is expressed on the surface of the activated CD4^+^ T cells. It converts immunoglobulins secreted by B lymphocytes to transformed classes by acting on CD40 molecules. Following mutations of the *CD40LG* gene, the expression of CD40L on the surface of T lymphocytes is reduced, and *CD40LG* cannot bind with associated molecules or affect the formation of CD40 trimers [16]. Thus, the CD40L gene defect constrains the interactions of T cells and B cells and affects germinal center formation [17]. To date, more than 250 unique mutations of the CD40L gene have been detected [16, 18]. A definitive diagnosis of HIGM requires the presence of decreased serum IgG along with one of the following: (1) a *CD40LG* pathogenic variant or (2) one or more maternally-related male members with an HIGM1 phenotype or diagnosis in the patient’s family. At this time, we reviewed all the seven patients who had been confirmed to carry a *CD40LG* mutation to reach a diagnosis of XHIGM (Table 1 and Fig. 1D). This patient presented a clinical disorder that began at the age of four months. At that time, the transplacental immunoglobulins from the mother would still contribute to maintaining the normal immune function of the infant [19]. However, the other six patients presented the first-onset clinical manifestation after the age of 18 months, when the maternal immunoglobulins were running out. As such, all six patients demonstrated a significantly increased level of IgM and decreased levels of IgA and IgG, which is different from the proband of this study. Taken together, infants with immune deficiency seldom suffered a critical infection attack, and it is difficult to distinguish the infants with immune deficiency from the patients with normal plasma immunoglobulin levels. Hence, exome sequencing is a powerful tool for seeking the comorbidities of bouts of severe infection in infants, especially for patients with a contributive family history.

Essential risk factors and comorbidities are usually present in young children with ARDS. Therefore, it is necessary to identify such related factors, which are mainly associated with clinical outcomes, for every case of ARDS. Accordingly, we attempted to identify any potential factors and comorbidities associated with ARDS development in this case. However, no sepsis, aspiration, trauma, drowning, or non-sepsis-related shock was uncovered. In very young infants, prematurity and low body weight are the most common underlying causes of ARDS. Moreover, congenital as well as acquired cardiovascular diseases, neurological disorders, cancer, and pre-existing pulmonary diseases also contribute to a poor ARDS prognosis. In light of the specific family history of this patient, we strongly suspected immune deficiency, even though the primary screening for plasma immunoglobulins yielded almost normal results. However, the proportion of immune cells had been recorded to be abnormal. Therefore, availing of exome sequencing, we identified XHIGM with the *CD40LG* mutation at a very early stage, without recurrent bouts of severe infection. The subsequent precise therapeutic approach enabled recurrent and opportunistic infections to be held at bay and prevented early death. Thus, once immunodeficiency is suspected in a patient at a very young age (< 6 months), exome sequencing is required after general immunological tests are completed.

Collectively, our experience with this patient emphasized the importance of early exome sequencing in identifying any potential risk factors for rare diseases or comorbidities, especially in a proband with a contributive family history. If the proband missed early exome sequencing, no curative medication could be administrated and there is a high risk of infant mortality. In such a condition, physicians and parents are usually unaware of the details of such patients, which means there is a risk of the same genetic variation occurring in the next offspring. Hence, early and timely exome sequencing for unexplained cases would not only benefit the treatment approach for the proband but also help to modify the strategy for the next reproduction based on PGT + IVF.

However, this case report still exists some limitations. Firstly, although the clinical and molecular results were sufficient to explain the phenotype of this proband, we only reported a single case that was much different from other patients in our center and in the literature. So, the conclusion should be handled carefully and seriously, Secondly, although several strategies had been employed to demonstrate the impaired function of the *CD40LG* c.346+ 1G > T mutation, experimental validation would be still required in the future.

In conclusion, ARDS is a severe condition among pediatric patients that is associated with significant morbidity and mortality rates. Several risk factors and comorbidities have been identified among pediatric ARDS patients with evident age-related characteristics. Immune deficiency is a common factor across age groups that contributes to the development of ARDS. As the incidence and clinical outcomes of ARDS are closely associated with a genetic disorder, it is critical to recognize immune deficiency at an early stage. Indeed, the *CD40LG* mutation is a hot topic, and hundreds of variants were identified during the last decade. However, we still consider this case report as having two strengths. (1) The c.346+ 1G > T is a novel mutation that was not reported before. (2) This is an infant who suffered an ARDS attack.

Usually, XHIGM would not be suspected with a relatively normal plasma immunoglobulin concentration. Also, exome sequencing helps target this patient's real facts over a limited period (2–3 weeks) very early. However, emerging evidence has demonstrated the importance of exome sequencing in identifying congenital disorders as a front-line consideration. It should be noted that general immunological tests should be performed to demonstrate the characteristics of the immune function at first, as the technical execution time limits exome sequencing to the very first-level examination. However, the priority of exome sequencing needs to increase among patients with unexplained infection attacks and the abnormal formation of subgroups of immune cells, especially for cases with a contributive family history among little-age infants, as the maternal transfused immunoglobulin might mask immune deficiency. Finally, we recommended a shift in the priority of exome screening to help identify the causes of unusual bouts of severe infection as general immunological tests could not fully explore certain types of deficiencies.

## Supplementary Information

**Additional file 1**. Supplementary materials.

## Data Availability

Data sets used in this study are available from the corresponding author upon reasonable request. The sequencing data has been deposited in NCBI (https://www.ncbi.nlm.nih.gov/bioproject/PRJNA847881).

## Abbreviations

ARDS: Acute respiratory distress syndrome
FACS: Fluorescence-Activated Cell Sorting
HIGM: Hyper IgM
HIV: Human immune deficiency virus
IVIG: Intravenous immune globulin
XHIGM: X-linked hyper IgM syndromes

## References

[1] Sapru A, Flori H, Quasney MW, Dahmer MK (2015). Pathobiology of acute respiratory distress syndrome. Pediatr Crit Care Med J Soc Crit Care Med World Fed Pediatr Intensive Crit Care Soc.

[2] Heidemann SM, Nair A, Bulut Y, Sapru A (2017). Pathophysiology and management of acute respiratory distress syndrome in children. Pediatr Clin N Am.

[3] Matthay MA, Zemans RL (2011). The acute respiratory distress syndrome: pathogenesis and treatment. Ann Rev Pathol.

[4] Ranieri VM, Rubenfeld GD, Thompson BT, Ferguson ND, Caldwell E, Fan E, Camporota L, Slutsky AS (2012). Acute respiratory distress syndrome: the Berlin definition. JAMA.

[5] Pediatric Acute Lung Injury Consensus Conference Group (2015). Pediatric acute respiratory distress syndrome: consensus recommendations from the Pediatric Acute Lung Injury Consensus Conference. Pediatr Crit Care Med J Soc Crit Care Med World Fed Pediatr Intensive Crit Care Soc.

[6] Khemani RG, Smith L, Lopez-Fernandez YM, Kwok J, Morzov R, Klein MJ, Yehya N, Willson D, Kneyber MCJ, Lillie J (2019). Paediatric acute respiratory distress syndrome incidence and epidemiology (PARDIE): an international, observational study. Lancet Respir Med.

[7] López-Fernández YM, Smith LS, Kohne JG, Weinman JP, Modesto-Alapont V, Reyes-Dominguez SB, Medina A, Piñeres-Olave BE, Mahieu N, Klein MJ (2020). Prognostic relevance and inter-observer reliability of chest-imaging in pediatric ARDS: a pediatric acute respiratory distress incidence and epidemiology (PARDIE) study. Intensive Care Med.

[8] Kitchin OP, Masekela R, Becker P, Moodley T, Risenga SM, Green RJ (2012). Outcome of human immunodeficiency virus-exposed and -infected children admitted to a pediatric intensive care unit for respiratory failure. Pediatr Crit Care Med J Soc Crit Care Med World Fed Pediatr Intensive Crit Care Soc.

[9] Gajic O, Dabbagh O, Park PK, Adesanya A, Chang SY, Hou P, Anderson H, Hoth JJ, Mikkelsen ME, Gentile NT (2011). Early identification of patients at risk of acute lung injury: evaluation of lung injury prediction score in a multicenter cohort study. Am J Respir Crit Care Med.

[10] Rowan CM, Smith LS, Loomis A, McArthur J, Gertz SJ, Fitzgerald JC, Nitu ME, Moser EA, Hsing DD, Duncan CN (2017). Pediatric acute respiratory distress syndrome in pediatric allogeneic hematopoietic stem cell transplants: a multicenter study. Pediatr Crit Care Med J Soc Crit Care Med World Fed Pediatr Intensive Crit Care Soc.

[11] Cortegiani A, Madotto F, Gregoretti C, Bellani G, Laffey JG, Pham T, Van Haren F, Giarratano A, Antonelli M, Pesenti A (2018). Immunocompromised patients with acute respiratory distress syndrome: secondary analysis of the LUNG SAFE database. Crit Care (Lond, Engl).

[12] Athipongarporn A, Ittiwut C, Manuyakorn W, Assawawiroonhakarn S, Larbcharoensub N, Shotelersuk V (2021). Diagnosis of hyper IgM syndrome in a previously healthy adolescent boy presented with cutaneous and cerebral cryptococcosis. Pediatr Infect Dis J.

[13] França TT, Leite LFB, Maximo TA, Lambert CG, Zurro NB, Forte WCN, Condino-Neto A (2018). A novel de novo mutation in the CD40 ligand gene in a patient with a mild X-linked hyper-IgM phenotype initially diagnosed as CVID: new aspects of old diseases. Front Pediatr.

[14] Flori HR, Glidden DV, Rutherford GW, Matthay MA (2005). Pediatric acute lung injury: prospective evaluation of risk factors associated with mortality. Am J Respir Crit Care Med.

[15] Wang LL, Zhou W, Zhao W, Tian ZQ, Wang WF, Wang XF, Chen TX (2014). Clinical features and genetic analysis of 20 Chinese patients with X-linked hyper-IgM syndrome. J Immunol Res.

[16] Jesus AA, Duarte AJ, Oliveira JB (2008). Autoimmunity in hyper-IgM syndrome. J Clin Immunol.

[17] Imai K, Slupphaug G, Lee WI, Revy P, Nonoyama S, Catalan N, Yel L, Forveille M, Kavli B, Krokan HE (2003). Human uracil-DNA glycosylase deficiency associated with profoundly impaired immunoglobulin class-switch recombination. Nat Immunol.

[18] França TT, Barreiros LA, Al-Ramadi BK, Ochs HD, Cabral-Marques O, Condino-Neto A (2019). CD40 ligand deficiency: treatment strategies and novel therapeutic perspectives. Expert Rev Clin Immunol.

[19] Longo NS, Lugar PL, Yavuz S, Zhang W, Krijger PH, Russ DE, Jima DD, Dave SS, Grammer AC, Lipsky PE (2009). Analysis of somatic hypermutation in X-linked hyper-IgM syndrome shows specific deficiencies in mutational targeting. Blood.

