# Supplementary material for: Exome sequencing contributes to identify comorbidities in a rare case of infant ARDS induced by the CD40LG mutation
Source: BMC Med Genomics. 2022 Jul 8;15:153. doi: 10.1186/s12920-022-01303-y (PMC9264746; doi:10.1186/s12920-022-01303-y)
Supplement: Supplementary file 1 — Additional file 1. Supplementary materials. [file 12920_2022_1303_MOESM1_ESM.docx]

**Genome Sequencing Contributes to Identify Comorbidities in a Rare Case of Infant (< 6 months) ARDS Induced by *CD40LG* Mutation**

Xue Gong et al.

**Additional file 1**

**Methods**

**1. Whole Exon Sequencing Analysis**

A peripheral blood sample was obtained using an EDTA anticoagulant blood sample tube, which was stored at 4°C for less than 6 hours. DNA was extracted using the Blood Genome Column Medium Extraction Kit (Tiangen, China) according to the manufacturer’s instructions. WES was performed using the Illumina NovaSeq 6000 platform (Illumina Inc. CA, USA), and the raw data were processed using Fastp for removing adapters and filtering low-quality reads. The paired-end reads were performed using the Burrows–Wheeler Aligner to the Ensembl GRCh38/hg38 reference genome. Variant annotation was performed in accordance with database-sourced minor allele frequencies (MAFs) and practical guidelines on pathogenicity issued by the American College of Medical Genetics.

**2. Mutation analysis of *CD40LG***

To understand the molecular architecture of the human *CD40LG* gene, we used MutationTaster with R software to predict the possibility of *CD40LG* c.346+1G>T to cause disease and its impact on protein structure. However, as the mutation of *CD40LG* c.346+1G>T located at the intron splicing motif and causing donor lost. SPIDEX (<http://tools.genes.toronto.edu)> has been used to predict the impact from this mutation on RNA splicing [1].

**3. Fluorescence-activated Cell Sorting (FACS)**

Peripheral blood mononuclear cells (PBMC) from the patient and his parents were isolated from fresh whole blood (P8670, Solarbio) and red blood cell were lysed (C3702-120, Beyotime). The lymphocytes were stained with CD3-FITC (ab34275, Abcam), CD8-PE (ab28017, Abcam) and CD40LG-APC (ab134406, Abcam). Fluorescence data were collected on a Propel Laboratories Avalon cytometer with a 70 μm nozzle. Data were further analyzed using FlowJo 7.0 software.

**References:**

1. Xiong HY, Alipanahi B, Lee LJ, Bretschneider H, Merico D, Yuen RK, Hua Y, Gueroussov S, Najafabadi HS, Hughes TR *et al*: **RNA splicing. The human splicing code reveals new insights into the genetic determinants of disease**. *Science (New York, NY)* 2015, **347**(6218):1254806.
